# A CRISPR interference system for engineering biological nitrogen fixation

**DOI:** 10.1128/msystems.00155-24

**Published:** 2024-02-20

**Authors:** Steven J. Russell, Amanda K. Garcia, Betül Kaçar

**Affiliations:** 1 Department of Bacteriology, University of Wisconsin-Madison, Madison, Wisconsin, USA; University of California San Diego, San Diego, California, USA

**Keywords:** *Azotobacter vinelandii*, CRISPRi, gene silencing, nitrogenase, nitrogen fixation

## Abstract

A grand challenge for the next century is in facing a changing climate through bioengineering solutions. Biological nitrogen fixation, the globally consequential, nitrogenase-catalyzed reduction of atmospheric nitrogen to bioavailable ammonia, is a vital area of focus. Nitrogen fixation engineering relies upon extensive understanding of underlying genetics in microbial models, including the broadly utilized gammaproteobacterium, *Azotobacter vinelandii* (*A. vinelandii*). Here, we report the first CRISPR interference (CRISPRi) system for targeted gene silencing in *A. vinelandii* that integrates genomically via site-specific transposon insertion. We demonstrate that CRISPRi can repress transcription of an essential nitrogen fixation gene by ~60%. Further, we show that nitrogenase genes are suitably expressed from the transposon insertion site, indicating that CRISPRi and engineered nitrogen fixation genes can be co-integrated for combinatorial studies of gene expression and engineering. Our established CRISPRi system fills an important gap for engineering microbial nitrogen fixation for desired purposes.

**IMPORTANCE:**

All life on Earth requires nitrogen to survive. About 78% of the atmosphere alone is nitrogen, yet humans cannot use it directly. Instead, we obtain the nitrogen we need for our survival through the food we eat. For more than 100 years, a substantial portion of agricultural productivity has relied on industrial methods for nitrogen fertilizer synthesis, which consumes significant amounts of nonrenewable energy resources and exacerbates environmental degradation and human-induced climate change. Promising alternatives to these industrial methods rely on engineering the only biological pathway for generating bioaccessible nitrogen: microbial nitrogen fixation. Bioengineering strategies require an extensive understanding of underlying genetics in nitrogen-fixing microbes, but genetic tools for this critical goal remain lacking. The CRISPRi gene silencing system that we report, developed in the broadly utilized nitrogen-fixing bacterial model, *Azotobacter vinelandii*, is an important step toward elucidating the complexity of nitrogen fixation genetics and enabling their manipulation.

## INTRODUCTION

All life requires nitrogen. Biological nitrogen fixation (N-fixation) reduces highly inert, atmospheric nitrogen (N_2_) to bioavailable ammonia (NH_3_) (1–3). This process has been driven by a unique enzyme, nitrogenase, for more than three billion years (4). Today, biological N-fixation is supplemented by the synthetic Haber-Bosch process, which currently sustains approximately half of the world’s population through crop fertilizer production (5–7). Our demand for fixed nitrogen is very high for food and agriculture. Despite its role in the agricultural boom of the 20th century, Haber-Bosch leads among chemical manufacturing in nonrenewable energy consumption and greenhouse gas emissions, responsible for nearly ~1% of carbon dioxide emissions caused by human activity (5, 8–10). Therefore, effective strategies to bioengineer nitrogen fixation are critically needed to lessen reliance on unsustainable industrial methods and to address nitrogen demand given its uncertain future in a changing climate (11–13).

The ability to modularly manipulate gene expression in N-fixing bacterial and archaeal “diazotrophs” would significantly expand the suite of synthetic biology tools for studying and engineering N-fixation. A detailed genetic understanding of biological N-fixation can speed the optimization of nitrogenase regulation and catalysis, the improvement of associations between diazotrophs and plants, and the transfer of N-fixation genes to cereal crops (6, 7, 14, 15). However, these efforts are stymied by a relative lack of sophisticated synthetic biology tools for diazotrophs (16).

**Fig 1:**
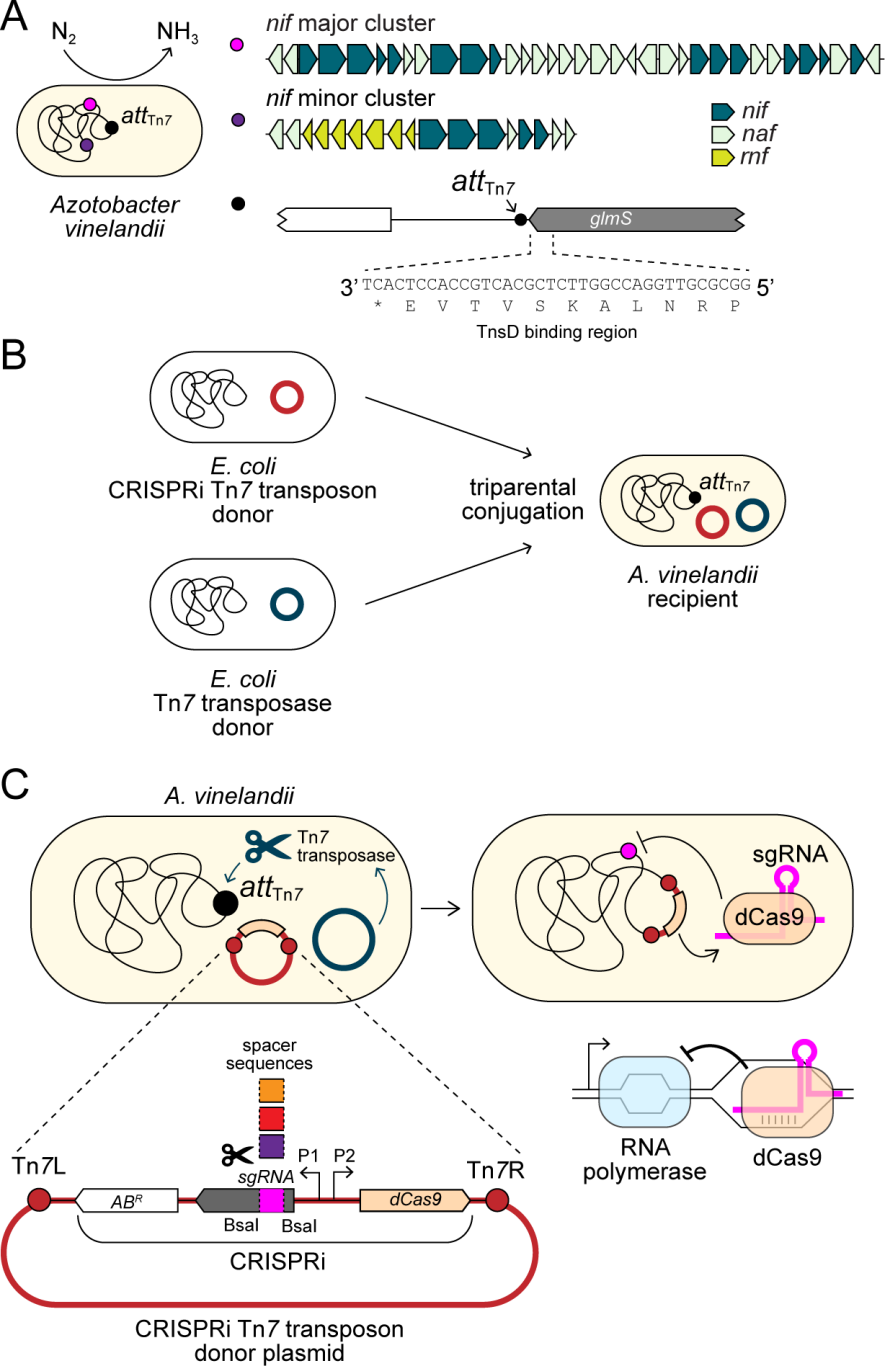
Integration and implementation of a CRISPRi system in *A. vinelandii*. (**A**) The diazotroph *A. vinelandii* possesses two clusters containing 52 genes (*nif*, *naf*, and *rnf*) that support the assembly and regulation of the molybdenum-dependent nitrogenase enzyme. The *A. vinelandii* genome also contains a 5′ *glmS* sequence motif that is highly conserved among gammaproteobacteria and serves as the TnsD binding region for downstream Tn*7* transposon insertion at *att*_Tn*7*_. (**B**) Mobile-CRISPRi plasmids are transferred to *A. vinelandii* via triparental conjugation. Two *E. coli* donor strains harbor plasmids with either the Tn*7* transposon containing the CRISPRi machinery (red) or the Tn*7* transposase machinery (dark teal). (**C**) The Tn*7* transposase enables genomic insertion of the CRISPRi transposon at the *A. vinelandii att*_Tn*7*_ site. Generalized schematic of the CRISPRi Tn*7* transposon is shown (lower left). Expression of dCas9 and sgRNA is controlled by separate inducible promoters of choice, P1 and P2 (P_L*lacO1*_ used in the present study; see Fig. 2). Unique spacer sequences are cloned into the Tn*7* transposon to program the knockdown of target genes within the *A. vinelandii* genome. The expressed dCas9 and sgRNA complex binds to the target region via base pairing between genomic DNA and the unique spacer sequence, interfering with transcript elongation by RNA polymerase (lower right).

Such is the case for one of the most prominent diazotrophic models, *Azotobacter vinelandii* (*A. vinelandii*), a free-living, aerobic gammaproteobacterium (17, 18) (Fig. 1A) for which gene mutation, gene knockouts (19–23), and transposon sequencing strategies have been applied to study N-fixation (24). However, no CRISPR/Cas9-based tools have been successfully leveraged in *A. vinelandii (16*) despite the wide applicability of this bacterium to agriculture and biotechnology (7, 18). Further, the functional roles and essentiality of many N-fixation-related genes under different physiological conditions have yet to be fully characterized (17).

A promising tool for the investigation and manipulation of N-fixation genetics in *A. vinelandii* is clustered regularly interspaced short palindromic repeats interference (CRISPRi) (25). This approach uses an RNA-guided, catalytically deactivated Cas9 nuclease (dCas9) to generate gene knockdowns by sterically blocking the transcription of target genes. dCas9 forms a complex with a single-guide RNA (sgRNA) and binds to the target gene via base pairing between the sgRNA and DNA. However, the mutant dCas9-sgRNA complex is incapable of cutting the bound DNA due to the loss of nuclease activity, and instead reduces total expression by preventing transcript elongation by RNA polymerase. The phenotypic consequences of gene knockdown can then be systematically assessed and/or leveraged for desired synthetic metabolic systems (26–30).

Here, we report the establishment of a CRISPRi system (25) for targeted N-fixation gene silencing in *A. vinelandii*, the first CRISPR/Cas9-based genetic tool for this bacterium. We discuss our results in the context of future applications of controlled gene silencing toward advancement of N-fixation bioengineering goals. Our system provides a critical foundation for much-needed advances in studies on the essentiality, the function, and the optimization of nitrogen fixation expression for desired applications.

## RESULTS

### CRISPRi machinery integrates into the *A. vinelandii* genome and achieves knockdown of target genes

To develop a CRISPRi system for *A. vinelandii*, we leveraged the Mobile-CRISPRi system, a suite of modular vectors for the conjugative transfer and genomic integration of dCas9 machinery into diverse bacteria (31) (Fig. 1). Development of CRISPRi for new hosts can be challenged by limitations to existing genetic tools for its transfer and incorporation, potential toxicity of expressed dCas9 or sgRNA [the latter resulting from off-target effects (32)], and poor knockdown efficiencies, all requiring testing and, if needed, additional optimization (27, 33). Mobile-CRISPRi, which includes components based on the *Streptococcus pyogenes* (*Spy*) dCas9 enzyme, broadens the transferability of CRISPRi to proteobacteria by leveraging the Tn*7* transposase system for genomic integration. Further, the modularity of its associated vectors enables improved specificity and optimization of CRISPRi parts. Mobile-CRISPRi was previously used to transfer CRISPRi components into several models (e.g., *Staphylococcus aureus*, *Listeria monocytogenes*, *Pseudomonas aeruginosa*, and *Zymomonas mobilis*) and non-model (e.g., *Vibrio casei*) proteobacteria and Firmicutes (27, 31). Thus, this approach is promising for the development of a CRISPRi system in the model gammaproteobacterium *A. vinelandii*.

We first assessed the feasibility of Tn*7* integration of CRISPRi machinery by identifying the Tn*7* transposon attachment site, *att*_Tn*7*_, in *A. vinelandii*, downstream of the *glmS* gene (Fig. 1A) (34). We confirmed the presence of a single *att*_Tn*7*_ site on the *A. vinelandii* chromosome. The 3′ end of *glmS* contains a sequence motif highly conserved across gammaproteobacteria that serves as a binding site for the transposase target selector protein TnsD (35). Further, the 3′ end of *glmS* in *A. vinelandii* is >500 bp from the nearest open reading frame, indicating that Tn*7* insertion would not likely disrupt downstream gene function and that *att*_Tn*7*_ would thus act as a neutral insertion site for CRISPRi components (36, 37).

**Fig 2:**
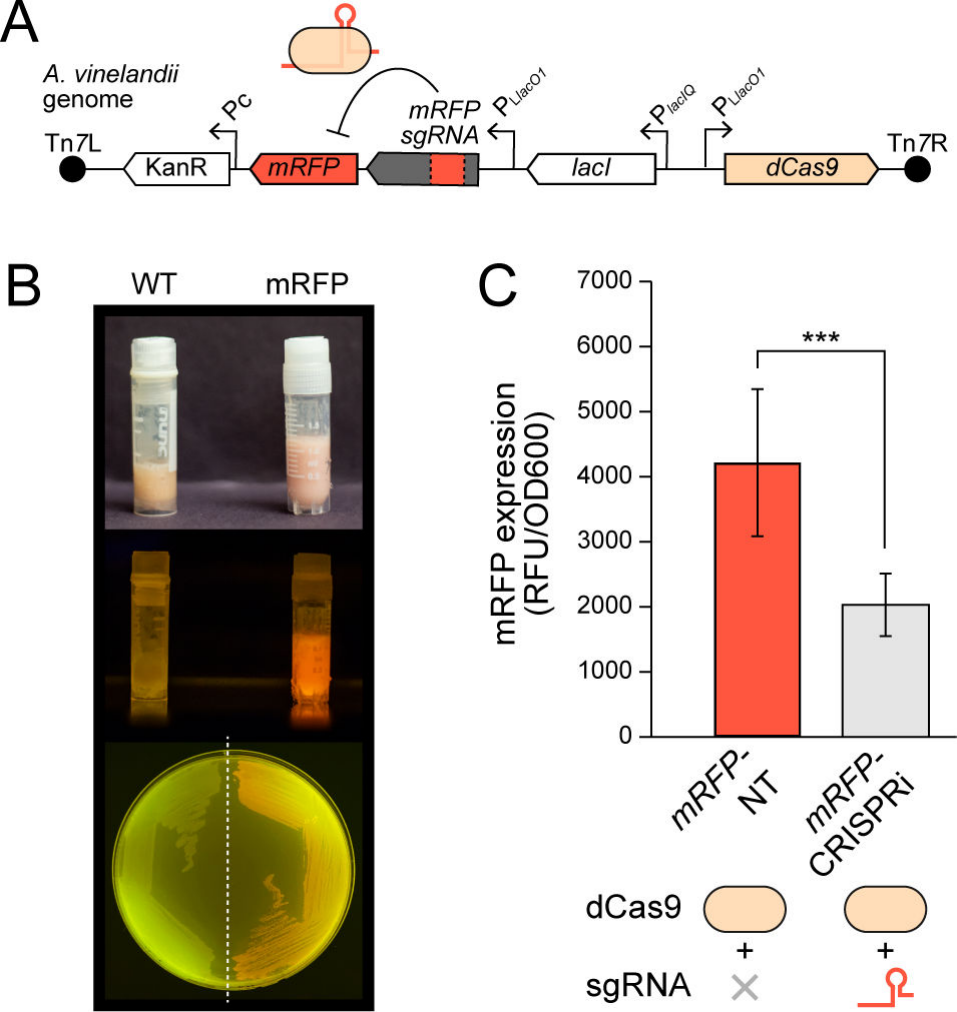
CRISPRi knockdown of a genomically integrated fluorescence reporter gene in *A. vinelandii*. (**A**) Schematic of the Tn*7* transposon inserted into the *A. vinelandii* genome, which contains *dCas9*, *mRFP*, and a sgRNA cassette with spacer sequence complementary to *mRFP*. (**B**) Images of *A. vinelandii* mRFP fluorescent phenotype from both strain stocks (top under natural light, middle under 465 nm blue light) and plated cells (bottom, under 465 nm blue light). Nonfluorescent WT strain shown for comparison. (**C**) Knockdown of mRFP expression in *A. vinelandii* cells assessed by fluorimetry. Fluorescence values are normalized to cell density (OD_600_) and corrected against baseline WT fluorescence (lacking *mRFP*). Each bar in the plot represents the mean of nine biological replicates with error bars indicating ±1 SD. ***: *P* < 0.001.

We attempted CRISPRi transfer to *A. vinelandii* using Mobile-CRISPRi test constructs that contain both CRISPRi as well as a fluorescence reporter cassette for initial determination of gene knockdown efficiency. These components include genes encoding *Spy* dCas9, monomeric red fluorescent protein (mRFP), sgRNA targeting *mRFP*, and a kanamycin antibiotic resistance marker (KanR) for Tn*7* insertion selection (33) (Fig. 2A). Both sgRNA and dCas9 expression are controlled by IPTG-inducible P_L*lacO1*_ promoters. Wild-type (WT) recipient *A. vinelandii* cells were mated with two *Escherichia coli* (*E. coli*) donor strains, one carrying a CRISPRi Tn*7* transposon vector and the other carrying a Tn*7* transposase vector (Fig. 1B). We isolated two *A. vinelandii* transconjugants, a “targeting” strain that possesses both *dCas9* and *mRFP*-targeting sgRNA (strain “*mRFP*-CRISPRi”) and a “non-targeting” strain with *dCas9* but lacking a sgRNA cassette (strain “*mRFP*-NT”) (Table 1). *mRFP-*containing strains were confirmed to exhibit a red fluorescent phenotype (Fig. 2B).

**TABLE 1:** Summary of *A. vinelandii* strains used and constructed in the present study (see Table S1 for additional details)

Name	nif	*att*_Tn*7*_	sgRNA target	sgRNA promoter	dCas9^*b*^	dCas9 promoter	Antibiotic resistance^*a*^	Parent	Source
DJ (WT)	*nifHDK*	n/a	n/a	n/a	n/a	n/a	n/a	n/a	Dennis Dean (Virginia Tech)
*mRFP*-NT	*nifHDK*	CRISPRi, *mRFP,* KanR	n/a	n/a	*Hsa Spy* dCas9	P_L*lacO1*_	KanR	WT	Present study
*mRFP*-CRISPRi	*nifHDK*	CRISPRi, *mRFP,* KanR	*mRFP*	PLlacO1	*Hsa Spy* dCas9	P_L*lacO1*_	KanR	WT	Present study
*nifH*-NT	*nifHDK*	CRISPRi, KanR	n/a	n/a	*Hsa Spy* dCas9	P_L*lacO1*_	KanR	WT	Present study
*nifH*-CRISPRi	*nifHDK*	CRISPRi, KanR	*nifH*	PLlacO1	*Hsa Spy* dCas9	P_L*lacO1*_	KanR	WT	Present study
DJ2566	*nifHDK*	n/a	n/a	n/a	n/a	n/a	StrR, GenR	n/a	Dennis Dean (Virginia Tech)
Δ*nif*	*ΔnifHDK*:: KanR	n/a	n/a	n/a	n/a	n/a	KanR, StrR, GenR	DJ2566	Present study
Tn*7-nif*	*ΔnifHDK*:: KanR	*nifHDK, lacZ*	n/a	n/a	n/a	n/a	KanR, StrR, GenR	Δ*nif*	Present study

^*a*^ KanR: kanamycin resistance cassette; StrR: streptomycin resistance cassette; GenR: gentamicin resistance cassette.

^*b*^ Hsa Spy dCas9 refers to the human codon-optimized *Streptococcus pyogenes* dCas9.

Gene knockdown efficiency was measured by fluorimetry of *A. vinelandii* cells following induction of the CRISPRi system targeting the co-integrated *mRFP* fluorescence reporter. Strain *mRFP*-CRISPRi cultured with IPTG (carrying both dCas9 and the *mRFP*-targeting sgRNA) showed an average 52% reduction relative to the non-targeting *mRFP*-NT strain (*P* = 0.0001) (Fig. 2C). Our results thus demonstrate successful induction of both dCas9 and sgRNA as well as repression of the *mRFP* target. Further, the growth of both targeting and non-targeting strains establish that neither dCas9 nor sgRNA expressed at the tested levels is toxic to *A. vinelandii*.

### CRISPRi knockdown reduces expression of proteins essential for N-fixation

**Fig 3:**
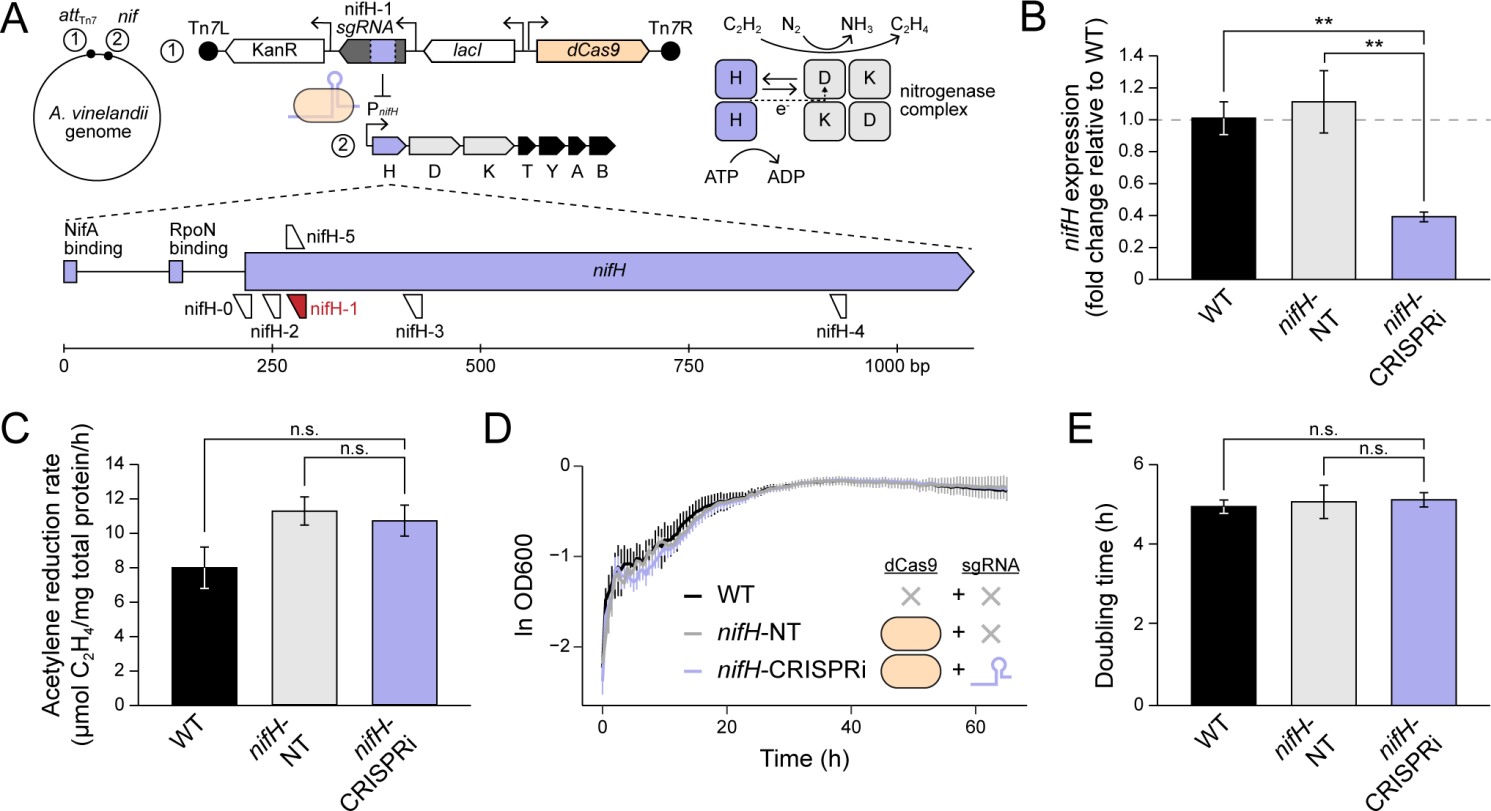
CRISPRi knockdown of native nitrogenase in *A. vinelandii*. (**A**) Schematic of the Tn*7* transposon inserted into the *A. vinelandii* genome containing *nifH*-targeting CRISPRi machinery. The simplified cartoon of the nitrogenase enzyme complex shows the arrangement of NifH (“H”, purple), NifD (“D”, gray), and NifK (“K”, gray) protein subunits. NifH protein, an essential component of nitrogenase, transfers electrons to the rest of the enzyme complex, ultimately resulting in the reduction of N_2_ to NH_3_ [as well as the alternate substrate acetylene (C_2_H_2_) to ethylene (C_2_H_4_)]. Five sgRNA spacer sequences (nifH-0 to nifH-5) complementary to genomic DNA between the P*_nifH_* promoter and the 3′ end of *nifH* were cloned into Tn*7* transposon plasmids. The construct containing sgRNA nifH-1 (red) was successfully transferred to *A. vinelandii*. (**B**) CRISPRi knockdown of *nifH* expression assessed by RT-qPCR. (**C**) Acetylene reduction rates of *A. vinelandii strains* following nitrogenase derepression. (**D**) Diazotrophic growth curve of *A. vinelandii* strains. Curves are averaged from three biological replicates per strain and error bars indicate ±1 SD. (**E**) Doubling times of *A. vinelandii* strains during diazotrophic growth. (**B, D, E**) Each bar represents the mean of three biological replicates, with the exception of that for WT in (**B**) and (**C**), which represents the mean of two biological replicates. Error bars indicate ±1 SD. **: *P* < 0.01; n.s.: not significant, *P* > 0.05.

We next tested CRISPRi knockdown efficiency of native nitrogen fixation genes encoding the nitrogenase enzyme in *A. vinelandii*. We designed sgRNA cassettes targeting the *nifHDKTY* operon, the latter which is under control of the *nifH* promoter (P*_nifH_*) and is expressed in the absence of a fixed nitrogen source in the culture medium (38–40) (Fig. 3A). This operon includes genes that code for the molybdenum-dependent nitrogenase enzyme complex (NifHDK), as well as the accessory nitrogen fixation proteins NifT and NifY, whose disruption was previously determined to have moderate or weak negative fitness effects in *A. vinelandii* (24). Under diazotrophic conditions, nitrogenase proteins are among the most highly expressed in *A. vinelandii* (39)—comprising ~10% of total protein (41)—creating a broad dynamic expression range for CRISPRi engineering.

We designed five sgRNA cassettes, each containing spacer sequences complementary to sense and antisense DNA strands located between promoter P*_nifH_* and the *nifH* 3′ end, the first gene within the *nifHDKTY* operon (Fig. 3A; Table 2). We designed multiple sgRNA sequences due to the previously demonstrated sensitivity of knockdown efficiency to the target binding region. For example, knockdown efficiency can vary depending on whether the spacer sequence is complementary to sense or antisense DNA strands (25, 42), and can also vary according to the spacer binding position across the length of the promoter and coding region (43, 44). Tn*7* transposon vectors, each cloned with one sgRNA cassette under the PLlacO1 promoter, were conjugated to WT *A. vinelandii* recipient cells. One construct, containing sgRNA “nifH-1,” targeting the antisense strand with +52 bp offset from the *nifH* start codon, was successfully transferred and inserted into *A. vinelandii*, yielding strain “*nifH*-CRISPRi.” A corresponding non-targeting strain, lacking a sgRNA cassette*,* was also isolated (strain “*nifH*-NT”).

**TABLE 2:** sgRNA sequences targeting knockdown of the *nifHDKTY* operon^*a*^

Name	Sequence	Sense	*nifH* start offset (bp)
nifH-0	TAGCCATAGTTAATTTCCTC	Antisense	−13
nifH-1	CACCAGGTTCTGAGTAGTGG	Antisense	52
nifH-2	GATACCACCTTTGCCGTAGA	Antisense	22
nifH-3	ATCTTCCACGGTACCGGCTT	Antisense	190
nifH-4	TCGTCGGCTTGCTTGGCTTT	Antisense	699
nifH-5	CACCACTACTCAGAACCTGG	Sense	50

^*a*^ Sequences bind sense or anti-sense DNA upstream of or within the *nifH* gene.

Expression knockdown of the *nifHDKTY* operon in strain *nifH*-CRISPRi was assessed by measuring *nifH* transcript levels by RT-qPCR under diazotrophic conditions, following induction of CRISPRi machinery by IPTG. We detected ~60% decreased *nifH* transcript in strain *nifH*-CRISPRi relative to both WT *A. vinelandii* (*P* = 0.009) and non-targeting *nifH*-NT strains (*P* = 0.003) (Fig. 3B). Our results demonstrate the successful repression of *nifH* by CRISPRi.

We further measured the phenotypic impact of *nifH* knockdown by characterizing diazotrophic growth and acetylene reduction activity following CRISPRi induction. Reduction of acetylene, an alternative substrate of the nitrogenase, to ethylene classically serves as a proxy for *in vivo* nitrogenase activity in *A. vinelandii* (45). Despite the ~60% decrease in *nifH* transcript detected by RT-qPCR, acetylene reduction rates of the same *nifH*-CRISPRi samples were comparable between WT (*P* = 0.055) and *nifH*-NT strains (*P* = 0.75) (Fig. 3C). Therefore, we find that total cellular nitrogenase activity is maintained in all strains despite the apparent reduction in *nifH* transcript for strain *nifH*-CRISPRi. Doubling times of all *A. vinelandii* strains under standard diazotrophic conditions were similarly comparable (*P* = 0.48; Fig. 3D and E), indicating that the achieved level of *nifH* knockdown did not significantly impact diazotrophic growth.

### Neutral expression of nitrogenase genes at the Tn*7* insertion site

After confirming CRISPRi knockdown of native nitrogenase genes, we designed a strategy to expand on the future utility of CRISPRi for investigations of N-fixation genetics by expressing nitrogenase genes from the Tn*7* insertion site. The Tn*7* site is typically neutral for insertion (i.e., does not result in a detectable phenotypic impact) (37), provided that the *att*_Tn*7*_ site does not disrupt neighboring genes (36), which we have not found to be the case in *A. vinelandii* (see above; Fig. 1A). Confirmation that nitrogenase genes can be successfully expressed from the Tn*7* site would permit the simultaneous incorporation of CRISPRi components targeting N-fixation genes as well as engineered or refactored N-fixation genes via Tn*7* insertion.

**Fig 4:**
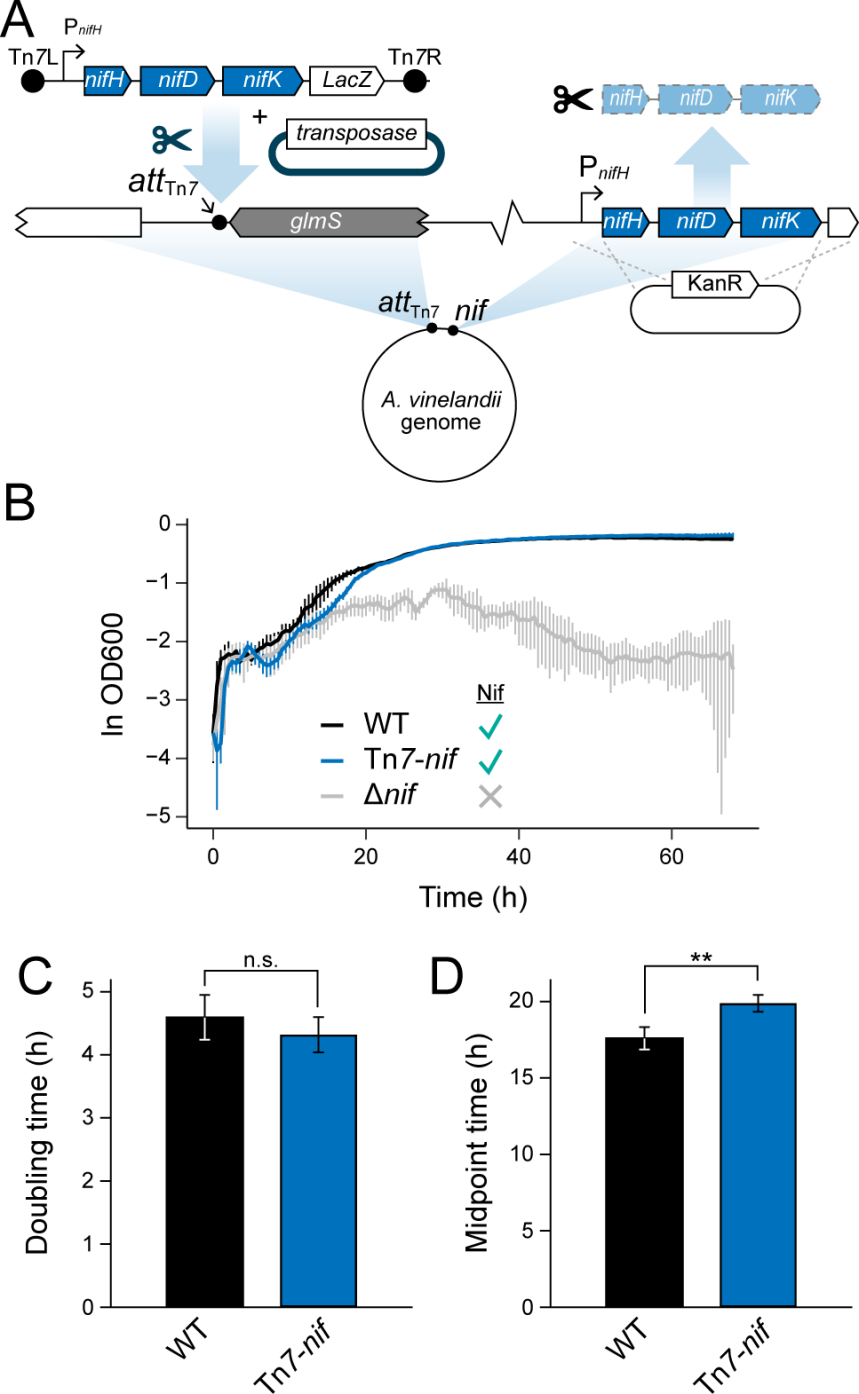
Neutral expression of nitrogenase *nifHDK* genes from the *A. vinelandii att*_Tn*7*_ site. (**A**) Relocation of native *nifHDK* genes to *att*_Tn*7*_ via Tn*7* transposon insertion. Native *nifHDK* was cleanly excised and replaced with a KanR cassette via homologous recombination. WT *nifHDK* genes under the P*_nifH_* promoter were cloned into a Tn*7* transposon and genomically integrated into the Δnif strain with Tn*7* transposase. (**B**) Diazotrophic growth curve of *A. vinelandii* strains. Curves are averaged from three biological replicates per strain and error bars indicate ±1 SD. (**C**) Doubling times of *A. vinelandii* strains during diazotrophic, exponential growth. (**D**) Midpoint times of *A. vinelandii* strains, representing the inflection point of a logistic model fit to the growth curve. (**C and D**) Each bar represents the mean of three biological replicates and error bars indicate ±1 SD. **: *P* < 0.01; n.s.: not significant, *P* > 0.05.

To test nitrogenase expression from the Tn*7* insertion site, a nitrogenase deletion strain, “Δ*nif*”, of *A. vinelandii* was first constructed, containing disruptions in all three *nif*, *vnf*, and *anf* gene clusters, the latter two encoding the alternative V- and Fe-only-dependent nitrogenases that is expressed under Mo-starvation (38) (Table 1). We then relocated *nifHDK* by inserting these genes back into the *A. vinelandii* chromosome by Tn*7* transposase at the *att*_Tn*7*_ site, generating strain “Tn*7-nif*” (Fig. 4A). The Δ*nif* deletion strain from which Tn*7-nif* was constructed exhibited severe defects in diazotrophic growth. These defects were not observed in prior growth in N-supplemented media, which was performed prior to inoculation and growth assessment in diazotrophic conditions (see Materials and Methods). Thus, the poor growth of Δ*nif* can be attributed to the removal of genes essential for nitrogen fixation. By contrast, Tn*7-nif* grew comparably to WT *A. vinelandii* under Mo-replete, diazotrophic conditions, with no significant difference in doubling time (*P* = 0.33) albeit a ~13% increase in growth lag time (*P =* 0.009) (Fig. 4B through D). Our results demonstrate that the reintroduction of *nifHDK* genes at the *att*_Tn*7*_ site of Δ*nif* is sufficient to rescue diazotrophic growth, and that expression of nitrogenase protein from *att*_Tn*7*_ is suitable for downstream N-fixation studies in *A. vinelandii*, including future co-integration of CRISPRi and nitrogenase components.

## DISCUSSION

The first functional CRISPRi system for the prominent, N-fixation model *A. vinelandii* reported here significantly expands upon available tools for the study and bioengineering of biological N-fixation. The complex nature of N-fixation genetics (46, 47) poses significant experimental hurdles and impedes predictions of how the N-fixation genetic network would respond to sequence-level engineering (39, 48), metabolic refactoring (49, 50), and differing environmental conditions. With so many targets, traditional genetic approaches for introducing mutations and gene deletions (via homology-directed recombination) are impractical for high-throughput analyses. *A. vinelandii* itself hosts 52 genes within clusters that support the regulation and maturation Mo-dependent nitrogenase system, as well as additional genes that support the alternative V- and Fe-only nitrogenases (17, 51).

This genetic complexity has been advantageous for N-fixation studies, providing source material for a wealth of scientific literature on nitrogenase enzymology (52–54), metallocluster assembly (20), regulation in response to fixed nitrogen source, metal availability, and nitrogenase mutations (38–40), electron transport to support the energy-intensive N-fixation pathway (55, 56), and coordination of the three variably metal-dependent nitrogenase systems (57, 58), including the Mo-dependent Nif nitrogenase studied here. In principle, these insights might be extended to other diazotrophs that contain subsets of N-fixation genes present in *A. vinelandii*, with the assumption that fewer genes represent a simplification of genetic schemes possessed by *A. vinelandii*, rather than altogether different schemes (48). Additionally, it has been suggested that the genetic complexity of diazotrophs like *A. vinelandii* has resulted from the optimization of N-fixation systems for an obligately aerobic bacterium (51). Thus, insights from *A. vinelandii* genetics are invaluable for understanding what is required to assemble and maintain the oxygen-sensitive nitrogenase in aerobic organisms, a significant hurdle for the goal of transferring N-fixation capability to cereal crops (6, 7).

A CRISPRi system for *A. vinelandii* presents several advantages over other genetic manipulation strategies that have previously been used to probe N-fixation gene function and essentiality. Both gene knockouts and transposon sequencing, the latter recently leveraged to quantify gene fitness effects in *A. vinelandii* (24)*,* are effective for identifying genes that contribute significantly to host fitness, but do not permit subsequent analysis of the phenotypic response if gene disruption proves lethal. Transposon sequencing avoids some of the disadvantages of traditional gene disruption methods by its high-throughput nature and is additionally not limited by effective selection markers. However, high sequencing coverage and the creation of large libraries are required (59). CRISPRi, like transposon sequencing, can permit the high-throughput characterization of N-fixation genes in *A. vinelandii* and other diazotrophs via the creation of sgRNA spacer sequences that enable targeted silencing of genes throughout the genome. Importantly, however, CRISPRi can be used to generate partial knockdowns and can be made to be inducible, titratable, and reversible (25, 31), allowing further characterization of essential gene response to varying levels of expression, and can be multiplexed to repress several genes simultaneously (60). CRISPRi systems have previously been introduced successfully into a variety of model and non-model organisms (25, 31, 59), including certain N-fixing bacteria and archaea [e.g., *Klebsiella pneumoniae* (61), *Methanosarcina acetivorans* (62), and *Rhodobacter capsulatus* (63)]. The utility of CRISPRi for N-fixation studies is now greatly expanded by its introduction into *A. vinelandii.* Importantly, genetic studies of *A. vinelandii* over the last 30 years have yielded an expansive catalog of mutant strains (17, 52, 64) amenable to further study with the tools presented here.

The modularity of the Mobile-CRISPRi vector suite we implemented can be leveraged to improve the efficacy of gene silencing in *A. vinelandii*, as well as overcome certain limitations and challenges associated with CRISPRi experiments. For example, development in new organisms can be hindered by toxicity of the dCas9 protein (65–67) and limitations on suitable sgRNA sequences. In our study, we demonstrate that these potential hurdles did not prevent development CRISPRi development in *A. vinelandii.* At least at the tested expression levels, we did not observe issues with *Spy* dCas9 toxicity in *A. vinelandii*, as strains with non-targeting CRISPRi components grew comparably to the WT control. Further, we show that effective sgRNA constructs can be leveraged in *A. vinelandii* without lethal or phenotypically detrimental off-target effects (32). Nevertheless, Mobile-CRISPRi constructs can be modified to incorporate different variants of dCas9 (68) or sgRNA designs, which may ameliorate unintended phenotypic effects or otherwise improve knockdown efficiencies. The 50–60% repression we observe, though within range of that previously achieved for N-fixation genes in other diazotrophs [from 50% to >90% (62, 69)], could be further optimized by placing CRISPRi components under more effective promoters for *A. vinelandii* and the testing of larger libraries of sgRNA constructs. Finally, prior work has shown that knockdown efficiencies can be fine-tuned by alteration of promoter strength (70), truncation of spacer sequences (71), or introduction of base-pair mismatches between the sgRNA and the target gene (72). We expect future work to expand on the utility of the CRISPRi system established here through such strategies and broader application to engineering N-fixation.

### Conclusions

We show that specifically designed CRISPRi components achieve targeted knockdown of both native and non-native genes, including those essential for the expression of the core N-fixing enzyme, nitrogenase. The *A. vinelandii* CRISPRi system, together with sequence-level manipulations of N-fixation components, provides an ideal groundwork for future, combinatorial investigations of N-fixation genetics, evolution, and bioengineering. In particular, such approaches could be used to tune N-fixation genetic network response to phylogenetically guided, protein-sequence-level engineering of nitrogenase proteins, which would expand the understanding and utility of nitrogenase catalytic diversity, both past and present (39, 48).

## MATERIALS AND METHODS

### Bacterial strains and culture conditions

All *A. vinelandii* and *E. coli* strains used or generated in the present study are described in Table 1; Table S1. *A. vinelandii* strains are derived from the DJ strain (ATCC BAA-1303; referred to here as wild-type, “WT”) generously provided by Dennis Dean (Virginia Tech).

*A. vinelandii* cells were cultured in liquid or solid Burk’s media (B medium) containing 1 µM Na_2_MoO_4_, with 10 mM ammonium acetate (providing a fixed N source, yielding “BN medium”), 1 mM IPTG, 0.6 µg/mL kanamycin, 100 µg/mL X-Gal, and/or yeast extract (Fisher Scientific Cat. No. BP9727; 5 g added per L of growth medium) amendments as needed (see below). Cells were grown at 30°C and liquid cultures were shaken orbitally at 300 rpm.

*E. coli* cells were cultured in liquid or solid Luria-Bertani medium (“LB medium”; Sigma Aldrich Cat. No. L3522), with 100 µg/mL carbenicillin and 300 µM diaminopimelic acid (DAP) amendments as needed (see below). The cells were grown at 37°C and liquid cultures were shaken orbitally at 250 rpm.

### Plasmid construction

Plasmids in the present study were obtained or modified from the Mobile-CRISPRi suite (31, 33) and are described in Table S2. Primers and synthetic oligonucleotides used for plasmid construction are listed in Table S3. pJMP1187 and pJMP1189 plasmids for testing Tn*7*-insertion of CRISPRi components and knockdown of the mRFP reporter in *A. vinelandii* are described previously and were generously donated by Jason Peters (University of Wisconsin-Madison) (31, 33).

Construction of plasmids targeting *nifH* knockdown followed Banta et al. (33) with modifications as described below. A sgRNA spacer sequence library for the *A. vinelandii* genome was generated via the build_sgrna_library.py code obtained from https://github.com/ryandward/sgrna_design. Five spacer sequences complementary to the genomic region between P*_nifH_* and the 3′ end of *nifH* were selected (nifH-0 to nifH-5). Single-stranded spacer oligonucleotides and their complements were synthesized with 4-bp BsaI overhangs (Integrated DNA Technologies, Coralville, IA, USA) and subsequently annealed. The resulting double-stranded DNA fragments were individually cloned into pJMP1339 following digestion with BsaI (New England Biolabs Cat. No. R3733), purification (Monarch PCR & DNA Cleanup Kit, New England Biolabs Cat. No. T1030), and ligation with T4 DNA ligase (New England Biolabs Cat. No. M0202), generating plasmids pSR38-pSR43 (see Fig. S1 for plasmid map of pSR39, containing sgRNA-nifH-1). A non-targeting plasmid, pSR44, was constructed by digestion of pJMP1339 with EcoRI to excise the sgRNA cassette followed by self-ligation (see Fig. S2 for plasmid map).

Plasmid pSR37 for Tn*7*-based insertion of *nifHDK* genes at the *A. vinelandii att*_Tn_*_7_* site was constructed by NEBuilder HiFi assembly (New England Biolabs Cat. No. E2621) from the pJMP6957 Tn*7* vector, lacking both CRISPRi and sgRNA cassettes (see Fig. S3 for plasmid map). A synthetic *lacZ* cassette (Twist Biosciences, South San Francisco, CA, USA) and a 4,653 bp *A. vinelandii* gDNA fragment containing *nifHDK* were PCR-amplified with primers 533/534 and 535/536, respectively, containing appropriate homologous sequences for HiFi assembly. pJMP6957 was digested with XhoI (New England Biolabs Cat. No. R0146) and purified (QIAquick PCR Purification Kit, Qiagen Cat. No. 28104) to excise the spectinomycin resistance cassette. The *lacZ* cassette was cloned into the pJM6957 plasmid via NEBuilder HiFi assembly, yielding plasmid pSR36. pSR36 was then digested with PmeI (New England Biolabs Cat. No. R0560) to excise the *sfGFP* cassette and assembled with the *nifHDK* fragment via NEBuilder HiFi assembly, generating plasmid pSR37.

All plasmid constructs were confirmed by whole-plasmid Oxford Nanopore sequencing (Plasmidsaurus, Eugene, OR, USA). Sequence-confirmed plasmids were transformed into *E. coli* mating strain WM6026 by electroporation for subsequent conjugation of *A. vinelandii* (see below).

### *A. vinelandii* strain construction

*A. vinelandii* mutant strains were constructed via Tn*7*-based genomic insertion of CRISPRi, antibiotic resistance marker, fluorescence reporter, and/or nitrogenase genetic components. Tn*7* transposon or transposase plasmids, hosted by *E. coli* mating strains (derivatives of WM6026), were transferred to *A. vinelandii* via tri-parental conjugation following Banta et al. (33), with modifications as described below. *A. vinelandii* parent cells were cultured in 50 mL BN medium to saturation (20–24 h). *E. coli* mating cells from two strains, the first harboring the desired Tn*7* transposon plasmid and the second, sJMP2954, containing the Tn*7* transposase plasmid pJMP1039, were cultured in 5 mL LB medium + DAP to saturation (16–18 h). About 100 µL of each of the three strains was combined with 700 µL BN medium with yeast extract (BNY medium), followed by centrifugation at 7,000 × *g* for 2 min. The resulting cell pellet was resuspended in 30 µL BNY medium, transferred to a nitrocellulose filter on solid BNY medium plates, and incubated at 37°C for 24 h. The nitrocellulose filter was then transferred to a 1.5-mL centrifuge tube and suspended in 200 µL 1× PBS, pH 7.4. Suspension aliquots were plated on appropriate selective solid medium: BN medium + X Gal for strain Tn*7-nif* and BN medium + kanamycin for all other *A. vinelandii* transconjugants. Plated cells were incubated at 30°C for 2–4 days. Transconjugants were screened by repeated scoring on selective solid medium and Tn*7* insertion was confirmed by PCR amplification of the *att*_Tn*7*_ site and Sanger sequencing with appropriate primers (Table S3; Fig. S4). All *A. vinelandii* transconjugants were additionally confirmed by Oxford Nanopore whole-genome sequencing (Plasmidsaurus, Eugene, OR, USA) (NCBI BioProject accession PRJNA1038772).

*A. vinelandii* strain Δ*nif*, the parent of strain Tn*7-nif*, contains disruptions to all three native nitrogenase gene clusters (*nif*, *vnf*, and *anf*) and was constructed from DJ2566 (inactivated for *vnf* and *anf* only; generously donated by Dennis Dean, Virginia Tech), following Dos Santos et al. (73). Competent DJ2566 cells were prepared via metal starvation (i.e., growth in BN medium lacking both Mo and Fe salts). Competent cells were transformed with ~1,000 ng of plasmid pAG25 containing a KanR cassette and flanking sequences directing the replacement of *nifHDK* genes by homologous recombination. Cells were plated on solid BN medium + kanamycin. Phenotypic screening and sequence confirmation of the *nifHDK* site (using appropriate primers; Table S3) were performed as described above.

### Fluorescence knockdown assay

Fluorescence assays followed Banta et al. (33) with modifications as described below. Seed cultures (nine biological replicates per strain) were inoculated from solid BN medium plates and grown in 50 mL liquid BN medium for 24 h. 1:100 dilutions of each seed culture were prepared in 5 mL BN medium followed by subsequent 1:100 dilutions in 50 mL BN medium + IPTG. The final dilutions were incubated for 10 doublings (24 h). About 1.5 mL of each culture was then pelleted and resuspended in 1.5 mL 1× PBS, pH 7.4. And 200 µL of each PBS suspension was aliquoted across a Nunc black-walled, flat-clear-bottom 96-well plate (Thermo Scientific Cat. No. 12-566-70), with five technical replicates each. OD_600_ and fluorescence (584 nm excitation, 607 nm emission) were measured by a CLARIOstar Plus Microplate Reader (BMG Labtech, Ortenberg, Germany). Fluorescence was reported as relative fluorescence units (RFUs) normalized to OD_600_ and statistical significance was assessed by an unpaired *t* test.

### Diazotrophic growth analysis

Diazotrophic growth characterization of *A. vinelandii* followed Carruthers et al. (74) as described below. Seed cultures (three biological replicates per strain) were inoculated from solid BN medium plates and grown in 50 mL liquid BN medium for 24 h. All strains grew comparably and reached a similar OD, including the Δ*nif* strain because growth in BN does not rely on nitrogenase expression and activity. The cells were then inoculated into liquid B medium (as well as B medium + IPTG for dCas9 and sgRNA induction in experiments with strains AK053 and AK054) to an OD_600_ of 0.05 and aliquoted across a 96-well, flat-bottom plate (Greiner Bio-One Cat. No. 655161), with three to five technical replicates each. Each plate was sealed with a Breathe-Easy gas-permeable, adhesive membrane (Diversified Biotech Cat. No. BEM-1) and incubated in a SPECTROstar Nano Microplate Reader (BMG Labtech, Ortenberg, Germany), which maintained 30°C internal temperature and 200 rpm double orbital agitation. OD_600_ measurements were obtained every 30 min for 3 days. Growth parameters were calculated using the R package Growthcurver (75) and statistical significance was assessed either by one-way ANOVA with post hoc Tukey HSD test or by unpaired *t* test.

### Acetylene reduction assay

*A. vinelandii* seed cultures (two biological replicates for WT and three biological replicates for other strains) were grown in 50 mL BN medium for 24 h. The cells were then inoculated into 100 mL BN medium + IPTG for dCas9 and sgRNA induction and grown to mid-log phase (OD_600_ = 0.5–0.8). Cultures were pelleted at 4,255 × *g*, 10 min at 4°C and resuspended in 100 mL B medium + IPTG for nitrogenase derepression and grown for 4 h [i.e., derepressed for nitrogenase expression by removal of a fixed N source (40)]. Rubber septum caps were affixed to the mouth of each flask and 25 mL of headspace was removed and replaced with an equivalent volume of acetylene gas via syringe. Cultures were subsequently shaken at 30°C, 300 rpm for 1 h, with headspace samples obtained every 20 min. Samples were analyzed by a Nexis GC- 2030 gas chromatograph (Shimadzu, Kyoto, Japan) and ethylene was quantified by a standard curve with known ethylene concentrations.

Immediately following extraction of the last headspace sample, 48 mL of each culture was pelleted at 4,255 × *g*, 10 min at 4°C and resuspended with 6 mL potassium phosphate buffer. About 1 mL aliquots of each resuspension was pelleted once more at 17,000 × *g,* 10 min and flash frozen in liquid nitrogen. Frozen pellets were stored at −80°C prior to total protein quantification and RNA extraction (see below). Total protein was determined using the Pierce BCA Protein Assay Kit (Thermo Scientific Cat. No. 23225) according to the manufacturer’s instructions on a CLARIOstar Plus plate reader (BMG Labtech, Ortenberg, Germany). Protein concentrations were quantified by a standard curve of known bovine serum albumin concentrations.

Acetylene reduction rates were calculated from three time points and normalized to total protein. Statistical significance was assessed by one-way ANOVA and post hoc Tukey HSD test.

### Nitrogenase gene expression analysis

Gene expression analysis was performed using flash-frozen cell pellets prepared from *A. vinelandii* culture samples immediately following their use in the acetylene reduction assay described above. These included three biological replicates per strain, with the exception of two biological replicates for WT. In addition, three cell pellets were collected per biological replicate. Cell pellets were stored for less than 1 week at −80°C prior to use.

RNA was extracted from each cell pellet (three pellets per biological replicate). Pellets were washed in 2 mL ice-cold 10 mM NaCl + 4 mL ice-cold RNAprotect Bacteria Reagent (Qiagen Cat. No. 76506) and repelletted at 4,255 × *g*, 10 min, 4°C. Pellets were resuspended in 200 µL TE buffer, pH 8.0 (Quality Biological Cat. No. 10128-420) with 50 mg/mL lysozyme (Ward’s Science Cat. No. 470301–618) and agitated at room temperature for 5 min. Column-based RNA extraction was performed on ice with the RNeasy Mini Kit (Qiagen Cat. No. 74104), according to the manufacturer’s instructions with additional on-column DNase I (Ambion Cat. No. AM2222) treatment (15 min at room temperature between column wash steps). RNA concentration and purity were assessed by a NanoDrop 2000 Spectrophotometer (Thermo Fisher Scientific, Waltham, MA, USA). *A*_260_/*A*_280_ ratios of the samples ranged between 2.04 and 2.08, and RNA concentrations ranged between ~800 and 1,800 ng/µL. RNA samples (three per biological replicate) were stored at −80°C for less than 24 h prior to use.

An independent RT-qPCR experiment was performed on each set of RNA samples (each set included one RNA sample per biological replicate; see above), with three RT-qPCR experiments performed in total. RT-qPCR was performed using the BRYT Green dye-based GoTaq 1-Step RT-qPCR kit (Promega Cat. No. A6020) with *nifH* (Locus tag Avin_01380) and reference gene 16S rRNA (Locus tags Avin_55000, Avin_55030, Avin_55060, Avin_55110, Avin_55140, and Avin_55170) primers reported by Poza-Carrión et al. (40) for *A. vinelandii* DJ (GenBank accession CP001157.1) (Integrated DNA Technologies, Coralville, IA, USA; Table S3). Reaction master mixes were prepared and 20 µL aliquots were distributed across a 96-well PCR plate (Bio-Rad Cat. No. MLL9601). Each 20 µL reaction mix contained 10 µL GoTaq qPCR Master Mix, 2× (from kit), 0.4 µL GoScript Reverse Transcriptase Mix, 50× (from kit), 200 nM forward primer, 200 nM reverse primer, 100 ng template RNA, and nuclease-free water. Mixes were prepared for biological replicates analyzed in duplicate alongside one no template control mix per primer set. The plate was sealed with adhesive Microseal “B” PCR Plate Sealing Film (Bio-Rad Cat. No. MSB1001). RT-qPCR experiments were run on a CFX Connect Real-Time PCR Detection System (Bio-Rad Cat. No. 1855201) following manufacturer’s recommended thermal cycling parameters for the GoTaq 1-Step RT-qPCR kit: 15 min reverse transcription at 37°C, 10 min reverse transcription and DNA polymerase inactivation at 95°C, and 40 cycles with each cycle comprising 10 s denaturation at 95°C, 30 s annealing at 60°C, and 30 s extension at 72°C steps. Primer specificity was assessed by the presence of single peaks in melt curve analyses. Cq threshold determination for each sample was performed with single-threshold and baseline subtracted curve fit options in the BioRad CFX Maestro Software v2.3. Cq values for no template controls were ≥28 (at least 11 cycles more than the experimental sample Cq range). Gene expression analysis was performed using the ΔΔCq method (76) relative to the 16S rRNA reference gene and WT control strain, with relative expression reported in terms of fold change. Fold change values across the three independent RT-qPCR experiments were averaged for each biological replicate prior to statistical analysis. Statistical significance was assessed by one-way ANOVA and post hoc Tukey HSD test.

## Data Availability

Genome sequences of *nifH*_CRISPRi, *nifH*_NT, and Tn7_*nif A. vinelandii* strains were deposited in the NCBI database (Bioproject accession no. PRJNA1038772). Additional data are available in the supplemental material or upon request.

## SUPPLEMENTAL MATERIAL

Supplemental Material msystems.00155-24-s0001.docx Supplemental figures and tables.

ASM does not own the copyrights to Supplemental Material that may be linked to, or accessed through, an article. The authors have granted ASM a non-exclusive, world-wide license to publish the Supplemental Material files. Please contact the corresponding author directly for reuse.
